# Supplementary figures and images for: Reconstruction of the molecular evolution of Usutu virus in Germany: Insights into virus emersion and circulation
Source: PLoS Negl Trop Dis. 2023 Oct 2;17(10):e0011203. doi: 10.1371/journal.pntd.0011203 (PMC10569574; doi:10.1371/journal.pntd.0011203)

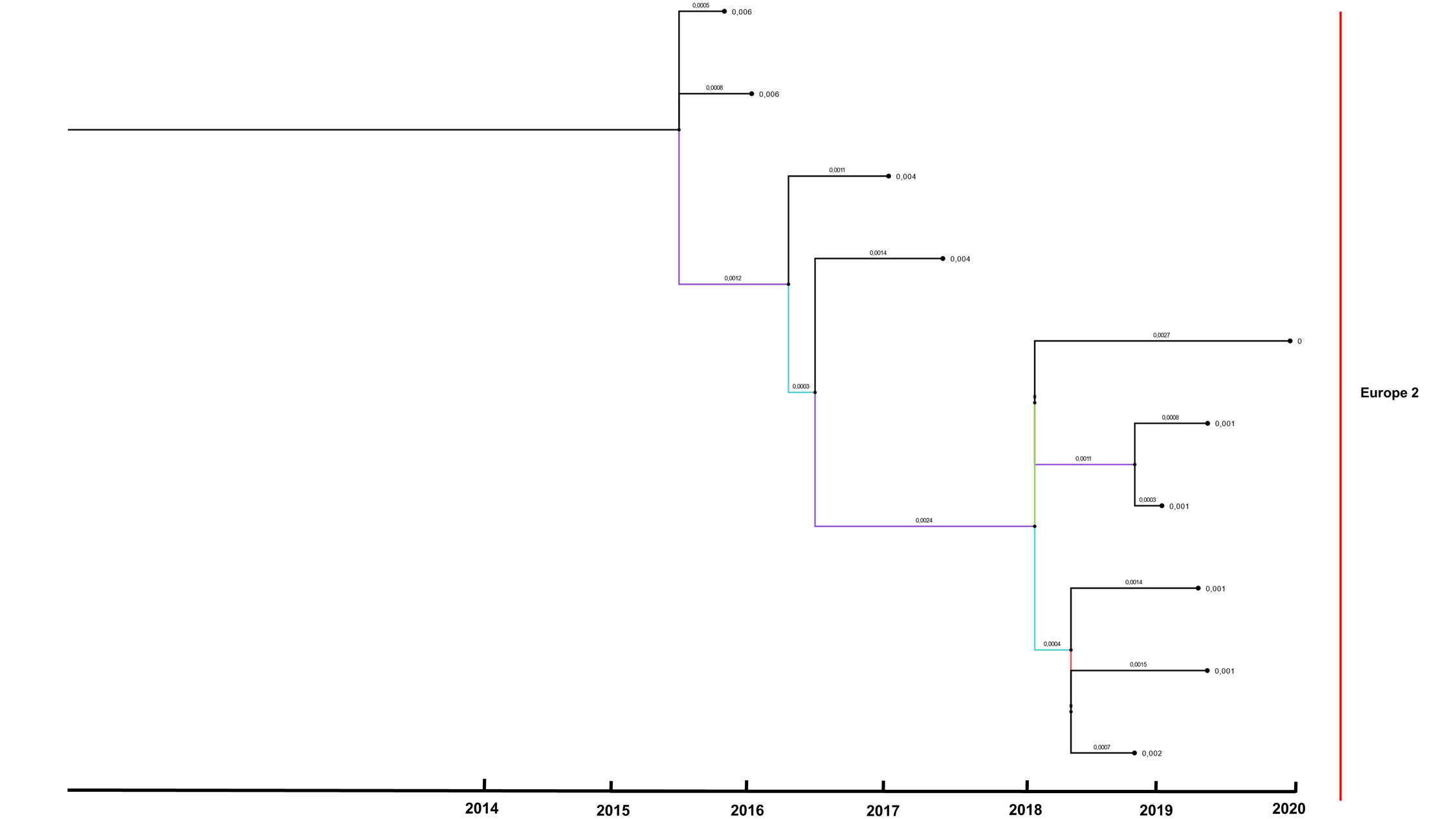

Supplement: S1 Fig — Node bars indicate 95% confidence intervals of the time of TMRCA. The branches are colored according to the sampling location of their nodes. (TIF) [file pntd.0011203.s002.tif]

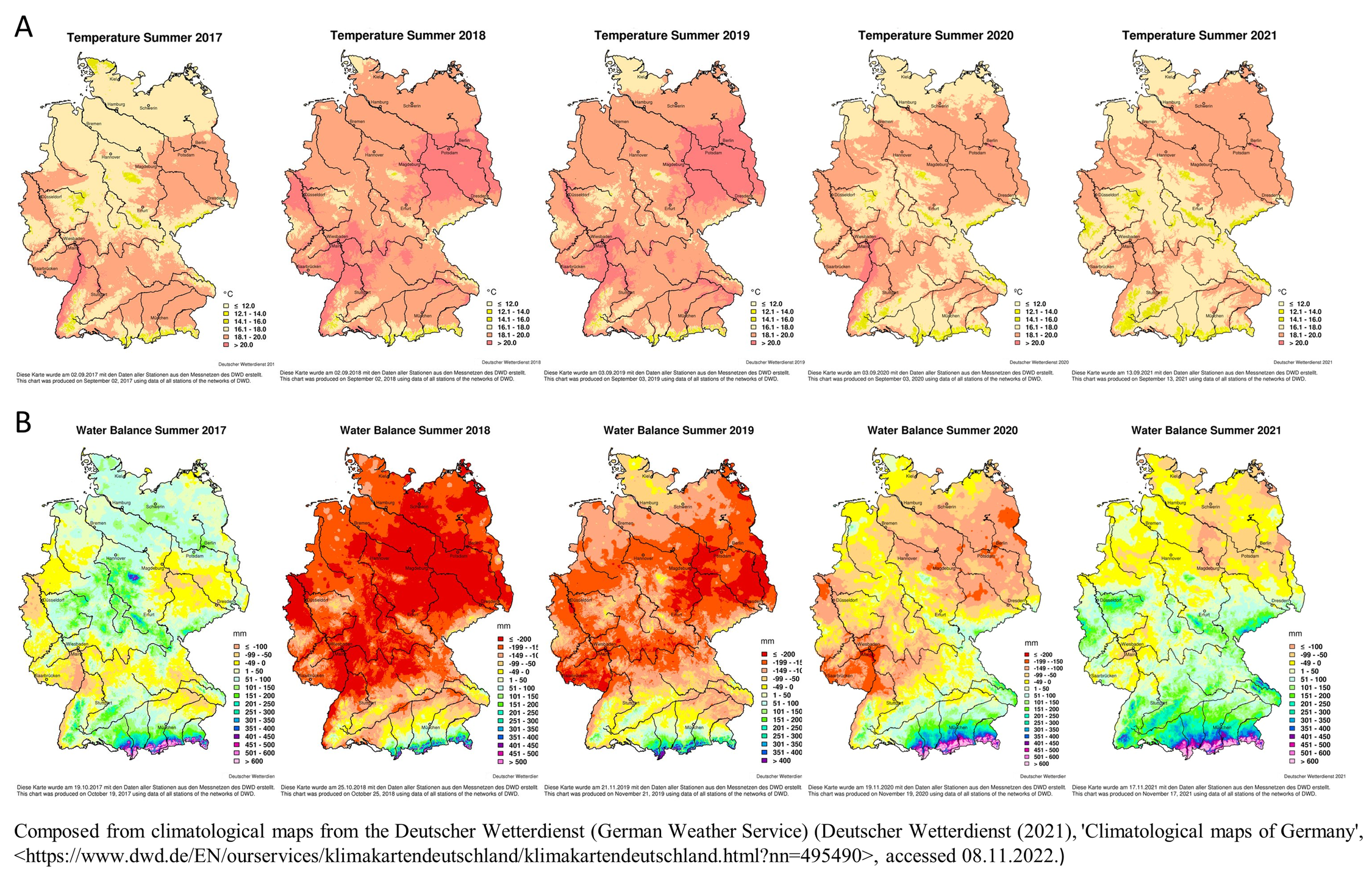

Supplement: S2 Fig — Climatological maps of Germany displaying (A) temperature (in degrees Celsius) and (B) water balance (in millimetre) based on data collected in the summers 2017–2021 [103]. Climatological maps were downloaded from the German Weather Service [104]. (TIF) [file pntd.0011203.s003.tif]

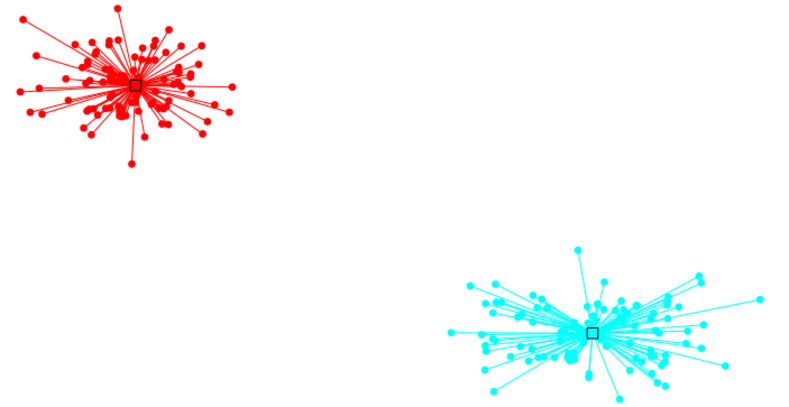

Supplement: S3 Fig — Each data point represents a viral sample, and the plot provides insights into the genetic diversity and distribution of the subclusters within the lineage. (TIF) [file pntd.0011203.s004.tif]

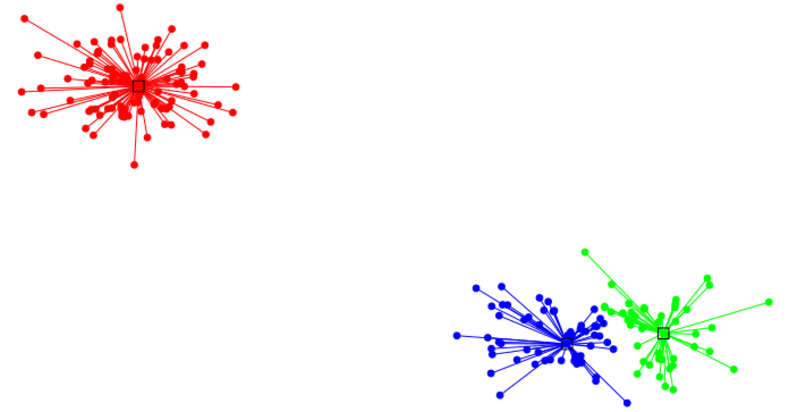

Supplement: S4 Fig — Each data point represents a viral sample, and the plot provides insights into the genetic diversity and distribution of the subclusters within the lineage. (TIF) [file pntd.0011203.s005.tif]
